# An unexpected ECG finding

**DOI:** 10.1007/s12471-015-0758-6

**Published:** 2015-10-08

**Authors:** Mathijs Kuiper, Albert Willems, Arthur A.M. Wilde

**Affiliations:** 1 Department of Internal Medicine, Sint Lucas Andreas Hospital, Amsterdam, The Netherlands; 2 Department of Cardiology, Haga Teaching Hospital, The Hague, The Netherlands; 3 Department of Cardiology, Sint Lucas Andreas Hospital, Amsterdam, The Netherlands; 4 Department of Cardiology, Academic Medical Centre, Amsterdam, The Netherlands

## Answer

**Fig. 1:**
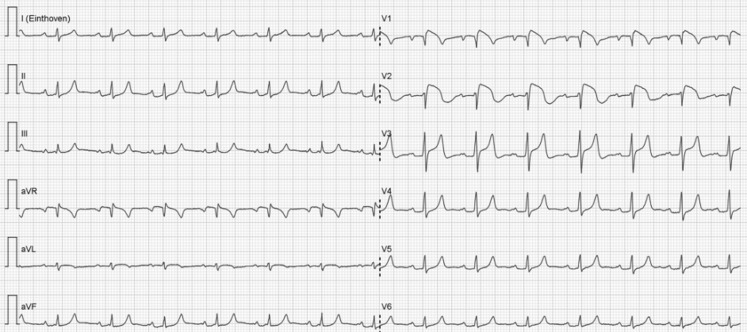
ECG at presentation

The ECG in Fig. 1 shows a sinus rhythm of 90 bpm with an intermediate axis, PQ delay of 202 ms and a narrow QRS complex. Convex ST elevation is seen in the right precordial leads. The ECG could be considered suspicious for acute septal myocardial infarction. However, reciprocal ST-segment changes are lacking and the QRS complex does not show any suspect abnormalities either. The ECG also has aspects of hyperkalaemia; in particular the sharp high voltage T waves point in that direction. The ECG in Fig. 1 is also compatible with a type 1 Brugada pattern. The patient has never had any cardiac symptoms, nor a family history of acute cardiac death.

**Fig. 2:**
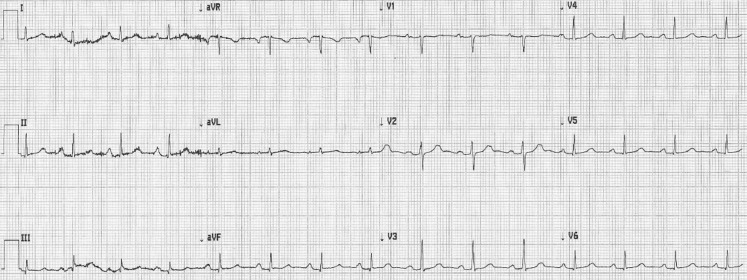
ECG after correction of hyperkalaemia

**Fig. 3:**
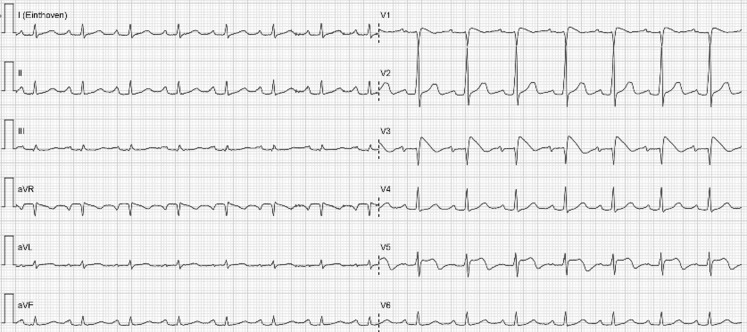
ECG during ajmaline provocation testing

The patient was admitted to the intensive care unit for rhythm monitoring. Hyperkalaemia was based on the use of high-dose spironolactone, in combination with dehydration and accordingly matched the patient’s comorbidity. Figure 2 shows the ECG taken after correction of the hyperkalaemia (5.2 mmol/l). This ECG returned to normal in a period of 4 h, without ST elevation and no signs of Brugada. Considering this short period of time to normalisation, there is a strong possibility that high potassium levels are provoking this Brugada pattern, as has been described before [1, 2]. Subsequently, Brugada syndrome was confirmed by positive ajmaline provocation testing. Shortly after administration of ajmaline the exact same type of Brugada was reproduced (with alternative placement of leads V3 and V5 to the intercostal space above V1 and V2, respectively; Fig. 3). This drug-induced Brugada syndrome is considered to be a type with a low risk of acute cardiac death [3]. However, in general it is recommended to prevent fever and if fever occurs to perform an ECG for potential rhythm monitoring, and to avoid certain drugs with the potential to prolong the QT interval.

## Conclusion

Hyperkalaemia-induced Brugada syndrome.

### Conflict of interest

None of the authors have any conflict of interest related to this report.
